# Structural Basis for the Specificity of Human NUDT16 and Its Regulation by Inosine Monophosphate

**DOI:** 10.1371/journal.pone.0131507

**Published:** 2015-06-29

**Authors:** Lionel Trésaugues, Thomas Lundbäck, Martin Welin, Susanne Flodin, Tomas Nyman, Camilla Silvander, Susanne Gräslund, Pär Nordlund

**Affiliations:** 1 Structural Genomics Consortium, Department of Medical Biochemistry and Biophysics, Karolinska Institutet, Stockholm, Sweden; 2 Division of Biophysics, Department of Medical Biochemistry and Biophysics, Karolinska Institutet, Stockholm, Sweden; 3 Chemical Biology Consortium Sweden, Science for Life Laboratories, Department of Medical Biochemistry and Biophysics, Karolinska Institutet, Solna, Sweden; 4 Centre for Biomedical Structural Biology, School of Biological Sciences, Nanyang Technological University, Singapore, Singapore; Instituto de Tecnologica Química e Biológica, UNL, PORTUGAL

## Abstract

Human NUDT16 is a member of the NUDIX hydrolase superfamily. After having been initially described as an mRNA decapping enzyme, recent studies conferred it a role as an “housecleaning” enzyme specialized in the removal of hazardous (deoxy)inosine diphosphate from the nucleotide pool. Here we present the crystal structure of human NUDT16 both in its apo-form and in complex with its product inosine monophosphate (IMP). NUDT16 appears as a dimer whose formation generates a positively charged trench to accommodate substrate-binding. Complementation of the structural data with detailed enzymatic and biophysical studies revealed the determinants of substrate recognition and particularly the importance of the substituents in position 2 and 6 on the purine ring. The affinity for the IMP product, harboring a carbonyl in position 6 on the base, compared to purine monophosphates lacking a H-bond acceptor in this position, implies a catalytic cycle whose rate is primarily regulated by the product-release step. Finally, we have also characterized a phenomenon of inhibition by the product of the reaction, IMP, which might exclude non-deleterious nucleotides from NUDT16-mediated hydrolysis regardless of their cellular concentration. Taken together, this study details structural and regulatory mechanisms explaining how substrates are selected for hydrolysis by human NUDT16.

## Introduction

Multiple non-canonical nucleotides appear as substrates and products in nucleotide metabolism, while multiple other damaged forms can be present as a consequence of various types of cellular stress. The appearance of elevated concentrations of any of these nucleotides may alter normal cellular function through competition with canonical nucleotides for binding to proteins as well insertion into RNA and DNA. Hence, life has developed two effective means of avoiding their hazardous effects: *i)* excision of the damaged base from the nucleic acids or *ii)* removal of damaged nucleotides from the nucleotide pool. The latter activity has been termed “housecleaning” [1], which means “to cleanse the cell of potentially deleterious endogenous metabolites and to modulate the accumulation of intermediates in biochemical pathways”.

NUDIX hydrolases belong to the category of “housecleaning” enzymes (NUDIX: nucleoside diphosphate linked to another moiety X). They are hydrolases that are specific for substrates composed by a **NU**cleoside **DI**phosphate linked to another moiety **X** [1]. Their actions result in the hydrolysis of a phosphodiester bond through a metal-assisted nucleophilic attack of a water molecule activated by a basic residue. The typical NUDIX reaction releases a (d)NMP and a phosphate, or a pyrophosphate, as products (NMP: nucleoside monophosphate). NUDIX hydrolases are characterized by the presence of the sequence element: GX_5_EX_7_REUXEEXGU, where U is a bulky hydrophobic residue and X can be any residue [1,2]. This motif is generally referred to as the NUDIX box and is located on a structural motif composed of an α-helix (the NUDIX helix) flanked by two loops. Chelation of the metal required for the catalysis is partially assured by Glu residues present in the NUDIX box. The deprotonation of the attacking water molecule is also often assisted by a Glu from the NUDIX box [3]. The NUDIX fold is composed of an α/β/α sandwich which may contain additional secondary structural elements depending on the enzyme specificity [4]. Their quaternary structure also varies among the different members of the NUDIX superfamily. NUDIX hydrolases are able to process substrates as different as the canonical (deoxy)nucleotides triphosphates [5], various forms of ADP-sugars [6,7], diadenosine polyphosphates [8], phospholipid precursors [6], NADH, Coenzyme A [9], capped mRNA [10], as well as altered nucleotides [4].

One member of this family, human NUDT16, has been initially described as a nucleolar RNA decapping enzyme specific for small nucleolar RNA in work based on its homology with *Xenopus laevis* orthologous, X29 [11]. Further studies conferred NUDT16 the role of a more general, nuclear and cytoplasmic decapping enzyme [12]. Its function partially overlaps the one of another NUDIX hydrolase, Dcp2, non-sense-mediated mRNA decay being mostly assured by Dcp2, while both decay of AU-rich-element containing mRNA and miRNA-mediated silencing can use either Dcp2 or NUDT16 [13]. More recently, the ability of NUDT16 to hydrolyze (d)IDP and (d)ITP has been shown in two complementary studies and thus conferred it an additional role in the “housecleaning” squad of enzymes [14,15]. Enzymatic studies show that NUDT16 can hydrolyze both inosine diphosphate (IDP) and its cognate deoxyribose (dIDP) into (d)IMP and Pi. (d)ITP, (d)GDP and XDP were also hydrolyzed by human NUDT16 although less efficiently. In the same studies, *NUDT16* gene silencing in HeLa cells correlated with accumulation of inosine in RNA as well as with an increase in single-strand breaks (SSB) in DNA. Additionally, this silencing led to the inhibition of the progression of HeLa cells. They remain stuck in the S phase of the cell cycle, probably induced by the high amount of single strand breaks (SSB) in genomic DNA [14]. Thus, similarly to inosine triphosphatase, ITPA, NUDT16 appears to play a role in maintaining low levels of (d)ITP through scavenging of the diphosphate form and thus takes part in the sanitization process of the nucleotide pool [16].

The most deleterious action of inosine-containing nucleotides is likely exerted following integration of dITP into DNA. This results in SSB through DNA repair mechanisms which can be further developed into double-strand breaks (DSB) [17]. An accumulation of either SSB or DSB eventually causes genetic instability and cellular death, thus explaining the toxicity of an elevated dITP pool [18,19]. Higher concentration of ITP, as well as the metabolic precursors, dIDP and IDP, might also have a negative impact on the activity of proteins using ATP or GTP or other purine nucleotides. Together this provides a rational why the savaging of this nucleotide pool is important for maintaining cell viability [20,21].

Here we report crystal structures of human NUDT16, both in its apo-form and in complex with IMP and Mg^2+^. This allowed the identification of critical determinants for substrate-recognition and suggests a catalytic mechanism for the (d)IDP hydrolysis reaction. Taken together with measurements of dissociation constants between NUDT16 and IMP, GMP and XMP, we can explain and quantitatively account for the role played by the different substituents on the purine ring in NUDT16/nucleotide interactions. While our results confirm those presented by Iyama *et al*. [14], the activity of NUDT16 towards ADP shows a significantly different profile suggesting that the carbonyl in position 6 on the base plays a critical role in the catalytic cycle of NUDT16. Finally, we identify and characterize potent product inhibition of NUDT16 by IMP, the binding of which prevents processing also of other substrates than (d)IDP. Taken together these data provide the first structural data for NUDT16 and allow us to propose an updated model of the mechanisms regulating NUDT16 activity in which its selectivity towards different substrates is not only regulated by the various affinities towards the substrates, but also by the intrinsic cellular levels of IMP.

## Results

### Crystal structure of NUDT16 (apo-form)

**Fig 1:**
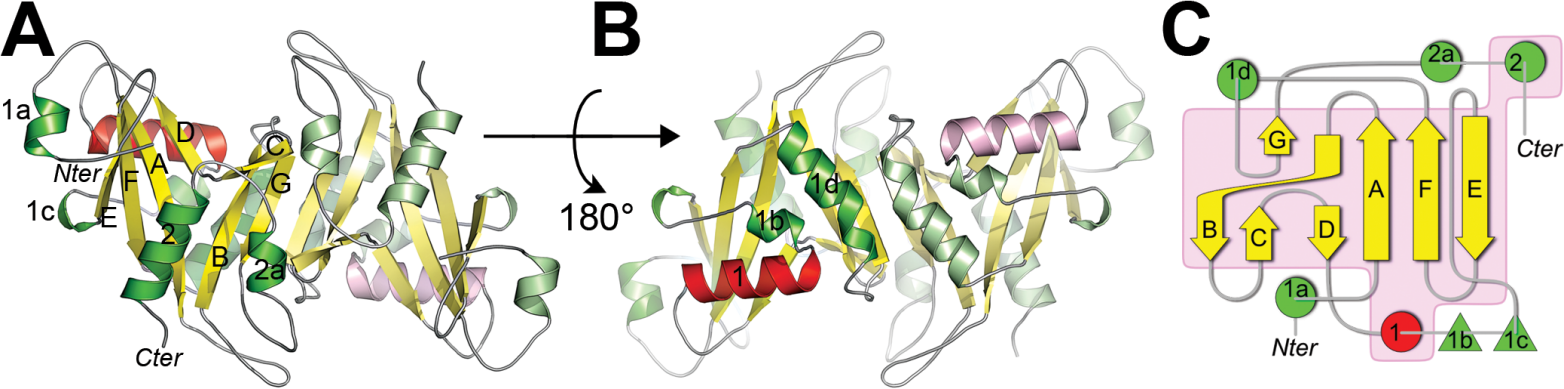
Overview of X-ray structure of apo-NUDT16 and sequence conservation of NUDT16 orthologous. (A and B) Apo-NUDT16 dimer. β-strands, helices and the NUDIX-box α-helix are shown in a cartoon representation and colored in yellow, green and red, respectively. Chain B, according to the PDB file, uses pastel colors. Secondary structure elements and location of the termini are labeled in black. (C) Topologic diagram of NUDT16. α-helices, 3_10_-helices and β-strands are shown as green discs, green triangles and yellow arrows, respectively with the exception of NUDIX helix 1 that is colored red. Secondary structure elements that are conserved among all NUDIX hydrolase structures are boxed in magenta.

One single NUDT16 monomer was found in the crystallographic asymmetric unit. Upon inspection of the interactions between symmetry-related molecules, it is clear that NUDT16 exists as a dimer (Fig 1A and 1B), which is common for NUDIX family members. Moreover, NUDT16 migrates in gel filtration similarly to a protein whose molecular weight is 1.7 times higher than NUDT16 monomer (S1 Fig). This observation and the analysis of potential protein-protein interfaces as determined by the PISA server confirm that NUDT16 is also present as a dimer in solution [22].

The secondary structural elements of the NUDT16 monomer are arranged according to the canonical NUDIX fold. The NUDIX-fold is an α/β/α sandwich which is constituted by two β-sheets; one being composed of two parallel β-strands (A and F) lined by two anti-parallel β-strands (D and E) and the second one by three anti-parallel β-strands (B, C and G). The two β-sheets interact through β-strands A and B in a way that the β-sheets could also be described as a single extended β-sheet.

Besides the secondary structure elements common to all NUDIX proteins, NUDT16 is decorated with five additional motifs (Fig 1A–1C). These supplementary structural elements are part of the hydrophobic core of NUDT16 (α-helices 1a, 2a, 2b and 1d; 3_10_ helix 1b) or serve roles to stabilize the NUDT16 dimer (α-helix 1a) or to maintain the orientation of the catalytic helix 1 (3_10_ helix 1c).

### NUDT16 dimerization mode

**Fig 2:**
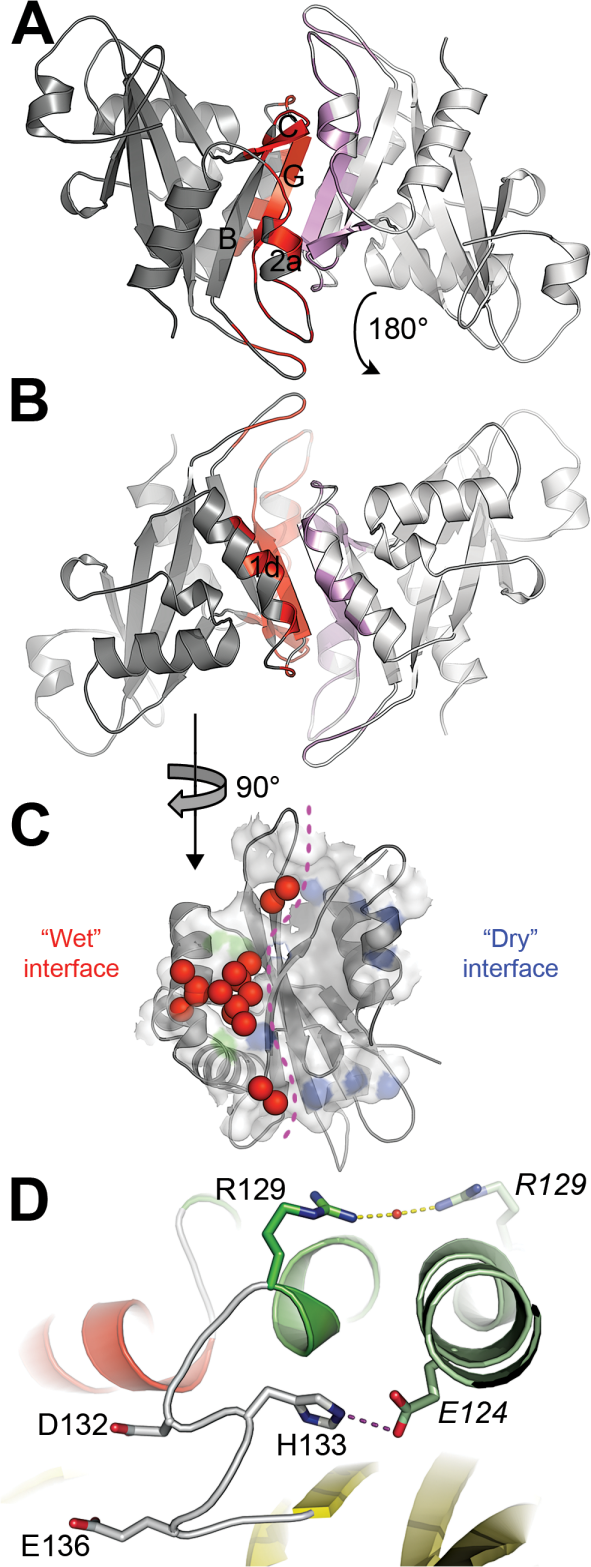
NUDT16 dimerization mode. (A and B) Apo-NUDT16 dimer. The two monomers are colored grey and white and regions participating to the dimerization process are colored red or pink whether they belong to the first or the second monomer. Views are identical to the one used in Fig 1A and 1B. Secondary structure elements involved in the dimerization process are labelled in black. (C) Dimerization surface. Only one monomer (the grey-colored one in Fig 2A and B) is represented as grey cartoons. Residues being buried after dimerization are shown as semi-transparent surface. Secondary structure elements involved in dimerization are shown as cartoons. Atoms involved in hydrogen-bonds and salt-bridges are colored blue and green respectively. Water molecules found in the interface are shown as red spheres. The border between the “wet” and the “dry” interfaces is represented by a violet dashed line. “Wet” and “dry” interfaces are located on the left and on the right side of the panel, respectively. (D) Role of α-helix 1d in the dimerization. Color code is identical to the one used in Fig 1A and 1B. Residues involved in polar interactions, Asp132 and Glu136 are shown as sticks. The water molecule bridging together the Arg129 from the two monomers is shown as a small red sphere. Ionic interactions and hydrogen-bonds are represented by magenta and yellow dashed lines, respectively. Residues are labeled using regular or italic characters depending whether they belong to the first or the second monomer, respectively.

The dimerization is mainly realized through interactions between the two β-strands G of two monomers which form, together with β-strands B and C, a six-stranded extended anti-parallel β-sheet (Fig 2A and 2B). The characteristics of homodimer interfaces are present in NUDT16 dimer with a buried surface area of 1384 Å^2^ per monomer and 27 residues involved per 1000 Å^2^ of buried surface [23]. Buried atoms are located in the loop between β-strands A and B, in β-strand C and in the region encompassing β-strand G and the two α-helices 1d and 2a (Fig 2C). NUDT16 dimer interface is subdivided into two regions: *i)* a “wet” interface whose contacts are mediated by α-helix 1d and β-strand G and *ii)* a “dry” interface comprising of α-helix 2a and the loop connecting β-strand B to β-strand C (Fig 2C). Noteworthy, all protein-protein H-bonds are mediated on the “dry” side of the interface and are organized as a ring around a large hydrophobic central region.

In addition to van der Waals interactions and hydrogen bonds between backbone atoms of β-strands G of the two monomers, a salt-bridge links Glu124 from each monomer to His133 of the other (Fig 2D). The consequence of these contacts is the stabilization of the position of His 133 in a cleft delimited by helix 1d and β-strand G. Such an orientation of His133 implies the repulsion of the two loop segments located on each side of His133 towards the NUDIX-helix 1. Because each of these segments comprises residues directly involved in metal-binding, the dimerization process in NUDT16 may increase the affinity towards metals and thereby improve the catalytic efficiency of NUDT16.

Besides its contribution to the buried surface area (14% of the total) upon dimerization, α-helix 1d also contributes in stabilizing the quaternary structure of NUDT16 through the formation of a water-mediated hydrogen-bond between the Arg129 of two monomers (Fig 2D).

### Interactions in the NUDT16 complex with product present

**Fig 3:**
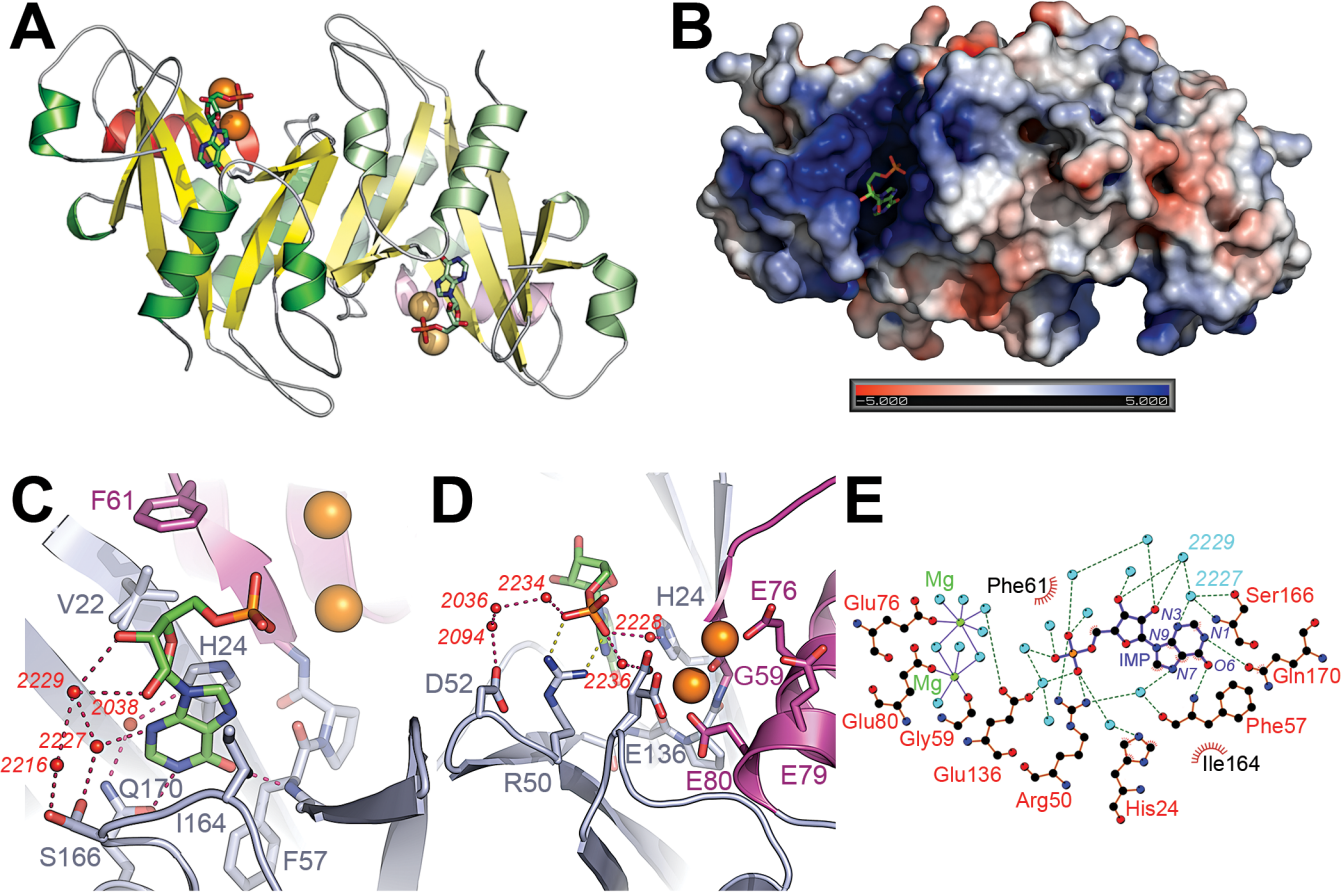
Interactions between NUDT16 and IMP. (A) Overview of the IMP molecules bound to NUDT16 dimer. Both the view and the color codes correspond to the ones used in Fig 1A. IMP and Mg^2+^ ions are shown as sticks and van der Waals spheres, respectively. (B) Electrostatic surface representation of NUDT16 bound to IMP. Molecular surface of NUDT16 is colored according to its electrostatic potential as calculated by the APBS program [24]. The color ramp varies from blue to red corresponding to +5*k*T/e and to -5*k*T/e, respectively. (C) Interactions between NUDT16, IMP and Mg^2+^ around the ribose and the base. NUDT16 is shown in the cartoon representation and colored either in violet (NUDIX motifs) or blue-grey (other region of the protein). IMP, IMP-interacting residues and Val22 are shown as sticks with their carbon atoms colored either in violet (residues belonging to the NUDIX motif), blue-grey (other NUDT16 residues) or green (IMP). NUDT16 residues are labeled using the single-letter code. Mg^2+^ ions are displayed as orange van der Waals spheres. Water molecules involved in hydrogen-bond network between NUDT16 and IMP are represented as small red spheres and labeled in red according to their numbering in the PDB file. H-bonds are symbolized by dashed lines. (D) Interactions between NUDT16, IMP and Mg^2+^ around the phosphate. Same color code as in (C) except that H-bonds and salt-bridges are represented by purple and yellow dashed lines respectively. (E) Ligplot [25] diagram of the interactions between NUDT16, IMP and Mg^2+^. Water molecules and Mg^2+^ ions are show as cyan and green spheres respectively. Covalent bonds in the IMP molecule are colored in violet and in orange when they belong to NUDT16. Hydrogen bonds and salt-bridges are represented by green dashed lines, hydrophobic interactions by red dashed lines. NUDT16 residues are labeled in black and red when they are involved in hydrophobic and polar interactions, respectively. Heteroatoms on the hypoxanthine ring of IMP are labelled in violet. Bonds between the Mg^2+^ ions and other atoms are depicted by violet plain lines. Water molecules (when they are shown in Fig 3C) are labeled in cyan according to their numbering in the PDB file.

While NUDT16 was incubated with IDP and Mg^2+^ prior crystallization trial (see Material and Methods), the structure contains a well defined product of the hydrolysis reaction, *i*.*e*. IMP (Fig 3A). In addition, the IMP-bound structure contains two Mg^2+^ ions. Except for some minor side-chain reorientations in the active site, no structural change is seen when comparing the apo- and ligand-bound structures.

It is worth noting that the protein construct used to obtain the two NUDT16 crystal structures harbors an A22V mutation (see Material and Methods section). A comparison of the active site of NUDT16 and X29 revealed that despite the presence of the A22V mutation, the structure of the active site region is well conserved between the two orthologous including the side-chain orientation of the two residues which are spatially closest of Ala22 (Phe61 and His24 in NUDT16) and therefore this mutation is not likely to effect the binding mode of the product. This was confirmed by comparing the kinetic parameters of NUDT16 wild-type with those obtained with the A22V mutation (S1 Table) when NUDT16 was assayed against XDP and ITP. The differences in the kinetic parameters caused by the A22V mutation are small (less than a factor 2), hence we considered that the A22V was a suitable model to study ligand-protein interactions. Detailed schemes of all interactions between protein, nucleotide and metals are shown on Fig 3C–3E.

The purine ring is buried in a hollow region of the protein (Fig 3B) whose electrostatic potential is highly positive, likely to accommodate the charges of the phosphate moieties carried by the substrates and the products. The cavity looks like a trench more than a pocket and this particular geometry might explain why NUDT16 is able to mediate the processing of both nucleotides [14] and small RNA molecules [26]. As a consequence, both the phosphate and the ribose ring are solvent-exposed in the IMP-bound structure, and this type of open architecture appears to be a prerequisite in order to accommodate extended substrates such as RNAs [12].

The cavity is mainly delimited by elements belonging to the NUDIX-fold common-core such as strands A, F, D, C, E and α-helix 2 (Fig 3A), although other secondary structure elements might help in selecting and orienting the substrate in the active site (see Discussion).

In the IMP-bound structure of NUDT16, the two Mg^2+^ ions have an octahedral coordination. Both metals are coordinated to one oxygen of a glutamic acid carboxylate of Glu80 and Glu76, respectively (Fig 3D). One of the Mg^2+^ ions is also coordinated to a main-chain carbonyl (Gly59) while the remaining coordination of the two Mg^2+^ ions is assured by water molecules, but where any of the two bridging water species might be hydroxide ions. All residues involved in metal coordination belong to the NUDIX motif.

Two phosphate oxygens interact directly with side-chain Arg50, which is a strictly conserved residue among NUDT16 orthologs and is involved in phosphate interactions in other homologs (Fig 3D and S2 Fig). All other interactions between the protein and phosphate atoms are mediated by water molecules (Fig 3D). Two of these water molecules make hydrogen bonds to His24 and Glu136, respectively. His24 and Glu136 also interact with the hypoxanthine base through a π-π interaction and with an active site Mg^2+^ through another water molecule-mediated interaction, respectively (Fig 3E). Asp52 connects to a phosphate oxygen through three water molecules (Fig 3D). This role, together with helping in the proper positioning of Arg50, may explain the strict conservation of Asp52 among NUDT16 orthologous (S2 Fig).

The two hydroxyl groups on the ribose moiety are connected to Ser166 Oγ (present in the NUDT16-IMP structure in dual conformation) through a network of water molecules (W2216, W2227 and W2229) (Fig 3C and 3E). No direct protein interaction involving the ribose moiety is seen in NUDT16-IMP structure. Like in the X29 structure, phosphate and the base moieties are making tight interactions while the ribose is only held in position loosely. The lack of direct interactions between the hydroxyl groups and protein residues might explain the lack of discrimination of NUDT16 between ribonucleotides and their deoxy counterparts [14].

**Fig 4:**
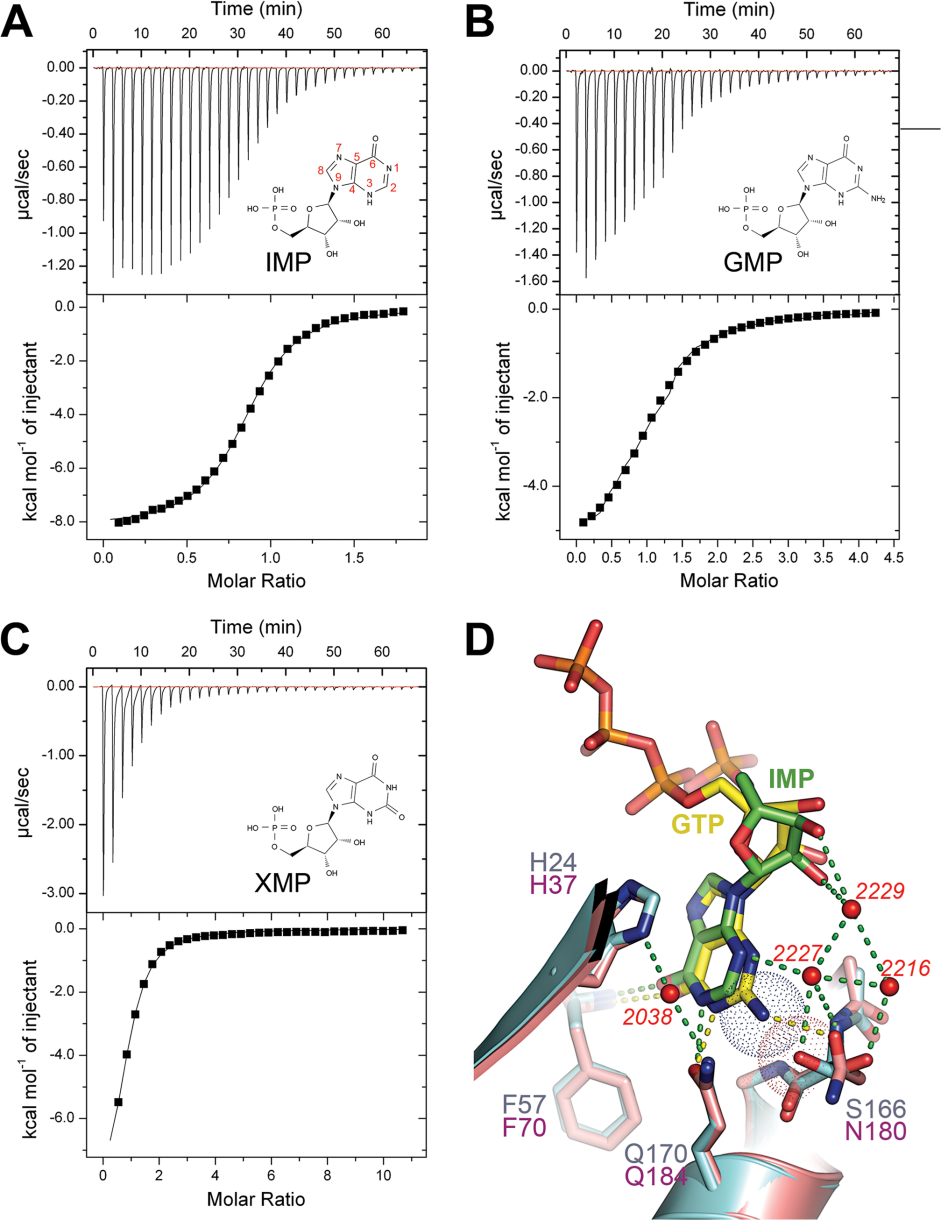
Isothermal calorimetry studies of NUDT16 with three products and structural basis for selectivity towards inosine containing nucleotides. (A,B and C) Isothermal calorimetry titration of IMP, GMP and XMP binding to NUDT16 respectively. The skeletal formulas of the different nucleotides monophosphates are represented in the chart displaying the raw data. Atoms on the purine ring are numbered in red on the IMP formula. (D) Superimposition between NUDT16 (cyan) and X29 (salmon) bound to IMP (green) and GTP (yellow), respectively. Residues involved in interaction and ligands are shown as sticks and labeled using the same color as the corresponding carbon atoms. Water molecules mediating hydrogen-bond network between NUDT16 and IMP are displayed as small red spheres and their position in the PDB file is presented in italic. Hydrogen bonds between NUDT16, X29 and their ligands are symbolized by green and yellow dashed lines, respectively. The van der Waals spheres corresponding to Ser166 Oγ and GTP N2 are represented by a red-dotted and a blue-dotted sphere, respectively.

Similarly to the structure of the complex between X29 and GTP, IMP is bound with its base in the *anti* conformation relative to the ribose ring while the C4’-C5’ bond is in a *gauche-gauche* orientation (Fig 4D). Three major interactions exist between NUDT16 and the base. As discussed above, the His24 side chain is involved in π-π stacking with the purine ring (Fig 3C). Furthermore, the hypoxanthine N1 and O6 atoms make hydrogen-bonds to Gln170 and Phe57, respectively. The critical role of these three direct protein/ligand interactions is highlighted by their high conservation in NUDT16 homologs (S2 Fig). The relative positions of the key residues His24, Gln170 and Phe57 are stabilized by additional polar interactions, which exist both in the apo- and ligand-bound forms of NUDT16. Hence, no significant structural rearrangements are apparent when IMP binds to NUDT16.

Besides these three conserved interactions, a water-mediated H-bond is observed between N3 on IMP and Ser166 Oγ (Fig 3C and 3E). In all NUDT16 orthologs, this position is occupied by residues that are able to serve as H-bond donors (S2 Fig). Ser166 is found in two conformations: one that is indirectly linked to the ribose ring through a water molecules network while the other has its side chain oriented towards the purine and mediates the above mentioned interaction with IMP N3. Only the first conformation exists in the apo-form of NUDT16, whereas the second is likely a consequence of product binding.

### Structural basis for substrate specificity

To establish an assay to look for product inhibition and to understand the role of the different substituents present on the purine ring, we first wanted to assess the activity of our protein preparations and compare it to the results obtained by Iyama *et al*. on NUDT16 activity on different nucleotides [14]. Contrary to the results presented in the structural biology part, we used wild-type NUDT16 (no A22V mutation) for these activity studies. Using a malachite green based activity assay for quantification of phosphate release, we determined the kinetic parameters of NUDT16 hydrolysis when incubated in the presence of ITP, XDP and ADP (S3A–S3C Fig), all of which have a suitable *K*_*m*_ for assessment with this assay and for inhibition studies (low μM range). The results are summarized and compared in Table 1 with the literature values. Using the same assay we have also confirmed that NUDT16 is able to process efficiently both GDP and IDP although the limited sensitivity of the malachite green assay does not allow us to determine sub-μM *K*_*m*_ values expected for these substrates (Table 1). Our *K*_*m*_ values for XDP and ITP are in the same range as data reported in Iyama *et al*. [14]. Discrepancies between the two studies can be explained by differences in the detection methods (HPLC or malachite green assay), the protein constructs (with or without His-tag) and the buffer conditions. Interestingly, the *K*_*m*_ for ADP was estimated to be about 30 times higher than for XDP suggesting unfavorable binding mode of this substrate. However, the *k*_*cat*_ for ADP was 15 times higher, leading to a catalytic efficiency in the same range as the one determined for XDP and ITP. Such a correlation between low affinity *K*_*m*_ values and low apparent rate constant values is also noticeable when looking more broadly at available data for the purine based substrates [14].

**Table 1:** Kinetic parameters of NUDT16 towards ITP, XDP and ADP and comparison with the results from Iyama *et al* [14]. *K*_m_, *k*_cat_ and *k*_cat_/*K*_m_ were determined by fitting the experimental kinetic data to a Michaelis-Menten model in GraphPad Prism version 5.01 for Windows, GraphPad Software, San Diego California USA, www.graphpad.com. The values indicated by the columns labelled “Ref. 14” are issued from the work of Iyama *et al* [14]. Substrates indicated with an asterisk (*) (IDP and GDP) are those whose low *K*_*m*_ values (according to Iyama *et al* [14]) are not suitable to have their kinetic parameters determined using malachite green assay.

	*K*_m_ (μM)		*k*_cat_ (min^-1^)		*k*_cat_/*K*_m_ (10^3^ s^-1^M^-1^)	
Substrate	This work	Ref. 14	This work	Ref. 14	This work	Ref. 14
IDP*	*n*.*d*.	0.066	*n*.*d*.	0.931	*n*.*d*.	251
GDP*	*n*.*d*.	0.33	*n*.*d*.	0.518	*n*.*d*.	26.1
ITP	8.23 ± 1.01	22.1	0.764 ± 0.030	3.06	1.55 ± 0.20	2.31
XDP	6.22 ± 0.55	15.7	0.368 ± 0.078	2.6	0.99 ± 0.23	2.76
ADP	185 ± 32	*n*.*d*.	5.754 ± 0.538	*n*.*d*.	0.52 ± 0.10	*n*.*d*.

In order to shed further light on effects of substituents on the purine, as well as potential product inhibition, we determined the dissociation constant of IMP, GMP and XMP to NUDT16 using isothermal titration calorimetry (Fig 4A–4C and Table 2). The highest affinity is seen for IMP leading to a dissociation constant of 5.24μM. The two other tested products bound NUDT16 with significantly lower affinities (*K*_*d*_ between 30 and 40μM). The affinity of AMP was too low for establishing good binding constants (data not shown).

**Table 2:** Binding constants of product binding to NUDT16. All values were determined by Origin from MicroCal Software, Inc., after fitting the experimental data to a one binding-site model, allowing all parameters to evolve freely in the course of the fit.

Product	*K*_*d*_ (μM)	N sites
IMP	5.24 ± 0.01	0.861 ± 0.003
GMP	34.25 ± 0.05	1.080 ± 0.015
XMP	39.06 ± 0.07	0.821 ± 0.024

The C6 carbonyl of IMP is involved in a 2.9 Å long H-bond with the amide main chain group of Phe57 (Fig 3C and 3E). If in adenine-containing substrates, the primary amino group in position 6 would occupy the position of the C6 carbonyl of IMP, and two H-bond donor groups would be forced in too close proximity. This would be energetically unfavorable. This is consistent with that the adenine moiety of m^7^GpppA in the X29 structure is present in a *syn* orientation in contrast to guanine and hypoxanthine found in *anti* conformation (S4A Fig). XDP and GDP, on the other hand, have a carbonyl in position C6, but also have a substituant in position C2 which leads to reduced affinity. This could be explained by either the disturbance of the water network mediated by Ser166 (Fig 4D) which connects N3 to the ribose ring or disturbance of the modes of resonance due to the polar interactions involving C6 and N1.

The orientation of the scissile phosphodiester bond between Pα and Pβ is very similar in the three mono- and triphosphate nucleotide complexes (S4A Fig). The work presented by Iyama *et al*. [14] revealed that NUDT16 *K*_*m*_ for IDP was 350 times lower than the one for ITP while *k*_*cat*_ was similar for both substrates. The structure of X29 in complex with GTP show that the Pγ sits in a position surrounded by many acidic residues (Glu136, Asp132, Glu79, Glu76 in NUDT16 numbering) [11]. While some are directly involved in interaction with Mg^2+^, the other ones might create an acidic patch likely to unfavor the binding of the triphosphate nucleotide compared to their diphosphate counterpart. The very similar *k*_*cat*_ on the other hand might be explained by the release of the IMP product being the rate limiting step for both substrates (see below).

### Catalytic mechanism

So far, the position of the catalytic base that activates the attacking water molecule remains elusive for members of this family. Based upon previous studies, Glu79, Asp132 or Glu76 have been proposed to play this role [3,11]. Glu89 (corresponding to Glu76 in human NUDT16) is involved in the simultaneous coordination of two manganese ions in the structure of X29 [11] (S4B Fig) and Asp132 is substituted by a tyrosine in *Nematostella vectensis* (S2 Fig). Thus, the only remaining candidate to act as catalytic base is Glu79 but this hypothesis needs to be confirmed by further studies (Fig 3D and S4B Fig).

A conformational change might occur in the binding site after the cleavage and the release of the first product (PPi or Pi) (S4B Fig). The two metals (labelled A and B in the figures), initially coordinated by Glu80, move towards Glu76 while Pα is displaced towards Arg50. In this scenario, while the negative charge of Pα is neutralized by Mg2+ A in the substrate, this role is conferred to Arg50 in the product/protein complex due to a rotation around the C4’-C5’ bond of the ribose. The displacement of the metal results in the rotation of Glu48 and in its engagement in a H-bond with Gln48.

### NUDT16 reaction is regulated by IMP inhibition

Due to the relatively low values of the dissociation constants of the complexes between NUDT16 and the products we measured using ITC, and the presence of the product in the crystals soaked with IDP, we wanted to assess the possibility that product inhibition would regulate NUDT16 activity. We therefore determined the IC_50_ of IMP when assayed against IDP, ITP, GDP, XDP and ADP. The results are summarized in Table 3.

**Table 3:** Inhibition by IMP. IC_50_ of IMP was determined by fitting for each substrate the normalized velocity to the dose-response model provided in GraphPad Prism version 5.01 for Windows, GraphPad Software, San Diego California USA, **www.graphpad.com**. As an example, a graph showing the influence of IMP on the activity of NUDT16 towards GDP is shown in S5 Fig. *K*_*i*_ was determined using the formula which links *IC*_*50*_ to *K*_*i*_ in the case of a competitive inhibitor, *K*_*i*_ = *IC*_*50*_/(1+[S]/*K*_*m*_) using either *K*_*m*_ values reported by Iyama et al. [14] (when the substrates used were IDP or GDP) or the ones we presented on Table 3 (for ITP, XDP and ADP).

Substrate	*IC*_*50*_ of IMP (mM)	*K*_*i*_ (μM)
IDP	3.174 (*R* ^*2*^ = 0.9532)	16.3
ITP	0.024 (*R* ^*2*^ = 0.9752)	9.8
GDP	0.795 (*R* ^*2*^ = 0.9711)	21.3
XDP	0.103 (*R* ^*2*^ = 0.9686)	35.2
ADP	0.006 (*R* ^*2*^ = 0.9692)	5.6

Measured IC_50_ values for different substrates were ranging from low micromolar to low millimolar. The wide range of IC_50_ values can be explained by the fact that we worked with the same concentration of substrates which have a wide range of *K*_*m*_. The experimental setup was selected due to the limitated sensitivity of the malachite assay. Based on the assumption that IMP acts as a competitive inhibitor, we calculate *K*_*i*_ for IMP using the values of IC_50_ we determined and the *K*_*m*_ values we (for ITP, XDP and ADP) or Iyama *et al*. [14](for IDP and GDP) have determined (Table 3). All determined *K*_*i*_ values lie in the low micromolar range confirming that the model we chose is appropriate and that IMP is effectively acting as a competitive inhibitor towards NUDT16 activity.

## Discussion

Here we present the crystal structures of apo- and IMP-bound human NUDT16, a metal-dependent hydrolase of the NUDIX superfamily. The structures reveal that the product (and by analogy the substrate) is accommodated in a conserved rigid binding-site. As many other NUDIX proteins, NUDT16 is a dimer. At the dimer interface, two loops connecting β-strand G to α-helix 2a and β-strand A to β-strand B respectively are projected over the ligand-binding site. This increases the depth of the active site and might improve the way capped RNA molecules approach NUDT16 and help in proper positioning of the nucleotide substrates in the active site. Moreover, the loop located between β-strand A and β-strand B, which also lines the dimer interaction surface, is rich in basic residues, and thus potentially increases the affinity of the NUDT16 towards negatively charged substrates (S2 Fig).

Comparison with the structure of the ortholog X29 in complex with various substrates allowed the identification of critical determinants for substrate specificity. A key aspect for the substrate is the presence of a carbonyl in position 6 (see nucleotide numbering in Fig 4A), in the IMP complex the inosine base making a hydrogen bond with the main chain amide of Phe57. Through comparison of m7GpppA and GTP bound X29, the kinetic profile obtained for NUDT16 assayed against ADP (high *k*_*cat*_ and *K*_*m*_ values compared to IDP, ITP, XDP and GTP), can be rationalized. A likely explanation for the high *K*_*m*_ is an unfavorable interaction with the Phe57 amine leading to the binding of adenine containing nucleotides in an *anti* conformation in contrast to the *syn* conformation seen for IMP and GTP. The high affinity of NUDT16 towards the carbonyl in position 6 will lead to low *K*_*m*_ and *k*_*cat*_ values in a catalytic profile where the rate-limiting step is likely the release of the monophosphate product, similarly as for MutT NUDIX hydrolase [27]. Hence, it is possible that among the NUDIX superfamily of proteins, the ones which contain the conserved motif GFP (S2 Fig), F standing for Phe57, will constitute a subfamily whose enzymatic profile might follow the same dependency as NUDT16 towards the carbonyl group in position 6. A second important determinant is likely to be the presence or the absence of a substituent in position 2 on the purine base. The NUDT16 structures support that a substituent in position 2 would hinder Ser166 to adopt a conformation where it engages in an elaborated network of H-bonds bridging the ribose ring, the base and the protein. An additional factor might be that the substituent in position 2 effects the induced resonance distribution of the purine ring upon binding, which might lead to less optimal polar interactions in the binding site.

Using the enzyme activity assay and binding studies with ITC, we provide data supporting that NUDT16 activity is highly regulated by IMP. The data is consistent with that the inhibition is competitive and that the binding constant is in the low micro-molar range. Due to its central role in the *de novo* synthesis of purine nucleotides, IMP concentration well into the micromolar range can be reached. There are significant variations in cell and tissue types, and in mouse for example IMP is hardly detectable in erythrocytes, readily detectable in liver, while muscle contains almost the same amount of IMP as ATP [28]. We therefore propose that the IMP inhibition of NUDT16 might provide a mean to regulate NUDT16 *in vivo* and to provide enhanced selectivity for IDP as a substrate. In the proposed regulatory mechanism, substrate specificity based on the affinity for the substrate (*K*_*m*_) is complemented by the *IC*_*50*_ of IMP for this substrate. In this scenario, inhibition mediated by mM levels of IMP would further attenuate NUDT16 activity towards non-hazardous substrates such as ADP and to a lesser extent GDP, while (d)IDP would be efficiently transformed to IMP. Thus, NUDT16 would scavenge deleterious (d)IDP without exhibiting a basal activity which will cause an imbalance of the nucleotide pool.

In summary, this structural and biochemical study has defined the critical determinants of substrate selectivity of NUDT16 for its NDP substrates (NDP: nucleoside diphosphate). Mechanistically, NUDT16 seems to be similar to MutT where the rate of the reaction is determined by the release of the monophosphate product, this product being also involved in the inhibition of the activity of the enzyme [27]. Such product inhibition potentially adds further IDP selectivity to NUDT16 and gives further support to the notion that this bi-functional protein might also play an important role in the scavenging of toxic inosine nucleosides in the cell.

## Material and Methods

### Cloning, expression and purification of NUDT16 for crystallography

The cDNA of human NUDT16 was purchased from the National Institute of Health's Mammalian Gene Collection (103) (accession no. BC031215). This cDNA harbors a mutation whose consequence is the replacement of Ala22 by Val in NUDT16 protein. The sequence encoding the full-length NUDT16 was amplified by PCR and subcloned into a pNIC28-Bsa4 vector (Novagen). Constructs are fused to a 6-His N-terminal tag followed by a Tobacco Etch Virus protease site.

NUDT16 expression plasmids were transformed into *Escherichia coli* BL21(DE3)-R3-pRARE2 strains provided by Structural Genomics Consortium (Oxford). The cells were grown in a LEX bioreactor system (Harbringer Biotechnology) in 750 ml of Terrific Broth (TB) media containing 8g/l glycerol and 50μg/ml kanamycin. Cell growth was performed at 37°C until OD_600_ reached 2.0, then cooled to 18°C and overnight gene expression was induced by addition of 0.5mM isopropyl β-D-thiogalactopyranoside. Cells were harvested by centrifugation, resuspended in lysis buffer (100mM Hepes (pH 8.0), 500mM NaCl, 10% glycerol, 10mM imidazole, 0.5mM TCEP) complemented with one tablet of complete EDTA-free protease inhibitor cocktail (Roche) and 1000 U benzonase (Merck), then frozen and stored at -80°C (TCEP: tris(2-carboxyethyl)phosphine, EDTA: Ethylenediamine tetraacetic acid).

Cells were disrupted by sonication then purified on a 1ml HiTrap Chelating HP column (GE Healthcare) equilibrated in buffer A (20 mM Hepes pH 7.5, 500 mM NaCl, 10% glycerol, 10 mM imidazole and 0.5mM TCEP). Bound NUDT16 was washed first in 20 column volumes of buffer A, then in 20 column volumes of buffer A containing 25mM imidazole instead of 10 mM. Protein was eluted in buffer A containing 500mM imidazole. The protein sample was subsequently loaded onto a Superdex S75 HiLoad 16/60 (GE Healthcare) equilibrated in buffer B (20 mM Hepes pH 7.5, 300 mM NaCl, 10% glycerol and 0.5 mM TCEP). Eluted fractions containing NUDT16 (as verified by SDS-PAGE analysis) were pooled together. Fresh TCEP at a final concentration of 2mM was added to the purified protein which was concentrated in a Vivaspin 20 (Sartorius) (cut-off 5000Da) concentrator to 30.1mg/ml (NUDT16). Samples were then frozen in liquid nitrogen and stored at -80°C.

### Cloning, expression and purification of NUDT16 for functional studies (isothermal titration calorimetry and activity assays)

The mutation V22A that was initially present in the purchased cDNA was reverted to the wild-type genotype by using the QuikChange Site-Directed Mutagenesis protocol (Stratagene). The plasmid described in the previous part was used as a matrix. Expression and purification procedures were identical to the ones used to obtain the sample for crystallographic studies with the following exceptions: the bacterial strain was *E*. *coli* Rosetta pLysS; the cultures were grown in a total volume of 1.5 l of TB in TunAir flasks; the affinity purification step was performed using 3x1 ml HisTrap HP columns (GE Healthcare) loaded with Ni^2+^ ions plugged in series and the gel-filtration column was a Superdex S200 HiLoad 16/60 (GE Healthcare).

After pooling the samples corresponding to NUDT16, the 6-His N-terminal tag was removed using Tobacco Etch virus (TEV) protease. An additional step of Ni^2+^-affinity purification using Ni-NTA agarose resin (Invitrogen) allowed the separation of cleaved NUDT16, His-tagged NUDT16 and TEV protease. The resin was equilibrated in buffer B supplemented with 10 mM imidazole. Cleaved NUDT16 was then dialyzed overnight at 4°C against buffer B. The protein was recovered and concentrated in a Vivaspin 20 (Sartorius) (cut-off 5000Da) concentrator to 24 mg/ml. Samples were then frozen into liquid nitrogen and stored at -80°C.

(Information concerning the procedures used to express and purify NUDT16 wild-type for analytical gel-filtration analysis and NUDT16 A22V for activity assay are described in S1 Text)

### Crystallization

#### Apo-NUDT16

Crystallization was performed using sitting-drop vapor diffusion at 4°C. 0.1 μl of protein was mixed with 0.1 μl of the reservoir solution containing 0.1 M Ches pH 9.5 and 20% w/v PEG8000 (condition A7 of JCSG+ screen (Qiagen)) and equilibrated against 50 μl of the reservoir solution. Crystals appeared in a week and were subsequently transferred to a solution of identical composition as the reservoir solution supplemented with 0.3 M NaCl and 25% glycerol prior to being flash frozen into liquid nitrogen.

#### IMP-bound NUDT16

NUDT16 was diluted to a concentration of 15 mg/ml in 20 mM Hepes pH 7.5, 300 mM NaCl, 10% glycerol and 2 mM TCEP. The protein solution was subsequently mixed with 2 mM inosine diphosphate (IDP) and 5 mM MgCl_2_. The sample was centrifuged (15min, room-temperature, 18000g) and the supernatant was used for crystallization trials. Crystals were grown using the sitting-drop vapour diffusion method at 4°C. 0.2 µl of NUDT16/IDP/MgCl_2_ was mixed with 0.1 μl of well solution containing 20 mM MgCl_2_, 0.1 M Hepes pH 7.5 and 22% polyacrylic acid (condition G2 of JCSG+ screen (Qiagen)) and equilibrated against 50 μl of the reservoir solution. After 75 days of incubation, crystals were harvested and briefly dipped into a cryo solution (25 mM MgCl_2_, 0.1 M Hepes pH 7.5, 24% polyacrylic acid, 2 mM IDP and 25% glycerol) and flash frozen in liquid nitrogen.

### Data collection, structure determination and refinement

Diffraction data for crystals obtained from apo-NUDT16 were collected at the BESSY beamline BL14.1. The crystallographic dataset corresponding to IMP-bound NUDT16 was collected at Diamond Light Source, on beamline I03. Data were indexed, integrated, scaled using XDS [29] and merged in SCALA [30,31]. Structures were determined by molecular replacement using PHASER [32]. Model based upon the structure of *Xenopus laevis* SnoRNA decapping protein X29 (PDB ID: 1U20) was produced by SWISS-MODEL [33] and used as search model for apo-NUDT16. Structure of IMP-bound NUDT16 was solved using the corresponding apo-structure as molecular replacement probe. The model of the apo-structure was improved by simulated annealing in CNS [34,35], followed by automated model building in ARP/wARP [36]. Final models were obtained after iterative cycles of manual model building in Coot [37] and maximum-likelihood refinement in REFMAC5 [31,38]. Data processing and refinement statistics are shown in Table 4.

**Table 4:** Data collection and refinement statistics.

	NUDT16 Apo form	NUDT16 IMP-bound
PDB Code	3COU	2XSQ
Data collection		
Beamline	BL14.1 (BESSY)	I03 (DIAMOND)
Wavelength (Å)	0.9795	0.9792
Space group	F23	F23
Cell dimensions		
a,b,c (Å)	142.13	141.58
α,β,γ (°)	90.0, 90.0, 90.0	90.0, 90.0, 90.0
Resolution (Å)	20.00–1.80 (1.90–1.80)	42.69–1.72 (1.81–1.72)
R_merge_ ^a^	0.067 (0.574)	0.061 (0.695)
R_p.i.m._ ^b^	0.030 (0.339)	0.025 (0.291)
<*I/σI*>	21.5 (2.5)	15.5 (2.3)
CC_1/2_	0.999 (0.764)	0.999 (0.779)
Unique reflections	22101 (3212)	25010 (3617)
Completeness (%)	99.9 (100.0)	100.0 (100.0)
Multiplicity	5.8 (3.8)	6.8 (6.6)
Refinement		
Resolution (Å)	19.71–1.80 (1.85–1.80)	42.69–1.72 (1.77–1.72)
R_work_/R_free_ (%)^c^	18.4/21.7	15.9/18.1
No. atoms		
Protein	1436	1420
Nucleotide	0	23
Metals	0	2
Water	196	236
Others^d^	0	1
B-factors (Å^2^)		
Protein	17.12	19.56
Nucleotide		19.24
Metals		26.65
Water	27.54	32.88
Others		53.20
Ramachandran plot (%)^e^		
Favorable	98.31	97.74
Allowed	1.69	2.26
Outliers	0.00	0.00
RMS deviations		
Bond lengths (Å)	0.018	0.010
Bond angles (°)	1.620	1.246

Values in parentheses refer to the highest resolution shell.

^a^ $R_{merge}=\sum_{hkl}\sum_{i}\left|I_{i}(hkl)-\left\langle I_{hkl}\right\rangle \right|/\sum_{hkl}\sum_{i}\left\langle I_{hkl}\right\rangle$

^b^ $R_{p.i.m.}={\sum_{hkl}\left[1/\left(N-1\right)\right]}^{1/2}\sum_{i}\left|I_{i}\left(hkl\right)-\left\langle I_{hkl}\right\rangle \right|/\sum_{hkl}\sum_{i}I_{i}\left(hkl\right)$

^c^ $R_{work}=\sum \left|\left|F_{obs}\right|-\left|F_{calc}\right|\right|/\sum \left|F_{obs}\right|$ where *F*_*obs*_ and *F*_*calc*_ are observed and calculated structure factors, respectively. R_free_ correspond to a subset of 5% of reflections randomly selected omitted during refinement.

^d^ Others refer to the Cl^-^ ion present in NUDT16 IMP-bound structure.

^e^ Values determined by MolProbity [39].

### Electrostatic surface potential calculation

Ionization states and atomic radii were calculated at pH 7.0 by PDB2PQR [40] using AMBER forcefield. Protonation state was determined by PROPKA [41] at pH 7.0. APBS [24] was then used to solve the linear Poisson-Boltzmann equation using the following parameters: solute dielectric: 2.0; solvent dielectric: 78.0; solvent probe radius: 1.4 Å; ionic strength: 0.15 M; temperature: 310 K. Mg^2+^ ions were included both in the calculation of the potential and in the surface representation presented on Fig 3B.

### Determination of kinetic parameters

Each sample point was obtained in the presence of 1 μM of NUDT16 (0.1 μM when assayed for activity against ITP) and various concentrations of substrates in buffer C (50 mM Tris-HCl pH 7.5, 150 mM NaCl, 20 mM MgCl_2_, 0.005% Tween-20, and 0.5 mM TCEP). When ITP was used as a substrate, 0.1 unit of yeast inorganic pyrophosphatase (Sigma-Aldrich) was added to the well. The activity assay was performed in 100 μl in a 96-well plate at 37°C and was stopped by addition of 25 μl of Malachite Green Working Solution (prepared as described in [42]). Plate was kept under shaking at room-temperature for 15 min, then 100 μl were transferred into a 384-well plate and the measurement of the absorbance at 630nm was carried out either on a CLARIOstar (BMG LABTECH) or a Victor^3^ (PerkinElmer) reader.

The amount of free phosphate produced during the hydrolysis was estimated by comparison with a standard curve.

Experiments were performed in triplicate and data analysis was done on GraphPad Prism version 5.01 for Windows (GraphPad Software, San Diego California USA, www.graphpad.com).

For each assayed substrate, starting substrate concentrations (varying from 0.5 to 240 μM) and incubation times (from 20 to 240 min) were selected in order for the quasi-steady-state assumption to remain valid.

The procedure was identical whether NUDT16 wild-type or A22V was used in the assay.

### Determination of IC50 of IMP against different substrates

For each substrate, the assay point is composed of 1 μM of NUDT16 (0.1 μM when assayed against ITP and IDP), 12 μM of substrate and various concentrations of IMP in buffer C. As described above the assay is complemented with 0.1 unit of yeast inorganic pyrophosphatase when the substrate is ITP. Assay conditions, free phosphate determinations and data fitting are performed as described in the previous section. Depending on the substrate, the range of IMP concentrations used in the assay varied between 0.05 μM and 325.8 mM. Experiments were performed in triplicate.

### Binding studies of products to NUDT16 using isothermal titration calorimetry

NUDT16 was dialyzed against 20 mM Hepes pH 7.5, 300 mM NaCl, 20 mM MgCl_2_, 10% glycerol, and 0.5 mM TCEP in a Slide-a-Lyzer cassette (7kDa cut-off). The calorimetric cell was filled with either 170μM (AMP-binding measurement), 180 μM (XMP-binding measurement), 200 μM (IMP-binding measurement) or 250 μM NUDT16 (GMP-binding measurement) while the syringe contained either 2 mM IMP, 5 mM GMP, 7.5 mM XMP or 13.4mM AMP. The buffer compositions in the cell and the syringe were carefully matched such that the sole difference between them was either the protein or the nucleotide content.

Titrations were performed at 25°C using 32 injections of 1.2μl of nucleotide solutions (following a 1μl pre-injection) spaced by 120 sec into the 200 μl experimental cell on a iTC_200_ MicroCal instrument (GE Healthcare) at the Karolinska Institutet/SciLifeLab Protein Science Core Facility (http://psf.ki.se).

Binding isotherm and thermodynamic constants were determined through fitting experimental data against a one-site binding-model (number of sites, ΔH and *K*_*a*_ being unconstrained during refinement) on Origin software v7.0552 (OriginLab).

## PDB accession numbers

The atomic coordinates and structure factors of NUDT16 (apo-form) and complex between NUDT16 and IMP have been deposited into the PDB under the accession codes 3COU and 2XSQ, respectively.

## Supporting Information

S1 Fig Analytical gel filtration of NUDT16 wild-type. (A) Overlay of gel filtration profiles. Protocol and elution volumes are presented in S1 Text. The chromatograms corresponding to standard proteins and NUDT16 are colored in black and red, respectively. The macromolecules used to generate the standard curve are abbreviated as such: Vit. B12 (vitamin B12), Apro. (aprotinin), Rib. A (ribonuclease A), C. A. (carbonic anhydrase), Ova. (ovalbumin), Con. (conalbumin), Bl. D. (blue dextran). (B) Calibration curve. The procedure used to determine the calibration curve and its equation is described in S1 Text. The equation obtained by linear regression and the coefficient of determination, *R* ^*2*^, are indicated in the graph area. The same abbreviations as in S1A Fig are used to identify the *K*_*av*_ values of the macromolecules used for the calibration.
(TIF)

S2 Fig Alignment between human NUDT16 and orthologuous. From the top to the bottom, the sequences used in the alignment correspond to the following GenBank IDs: 285026434 (*Homo sapiens*), 313231008 (*Oikopleura dioica*), 198435502 (*Ciona intestinalis*), 156393910 (*Nematostella vectensis*), 528517610 (*Danio rerio*), 410898918 (*Takifugu rubripes*), 432864527 (*Oryzias latipes*), 260810044 (*Branchiostoma floridae*), 284813508 (*Xenopus (Silurana) tropicalis*), 327264231 (*Anolis carolinensis*), 340368437 (*Amphimedon queenslandica*), 556107045 (*Lottia gigantean*), 242004576 (*Pediculus humanus corporis*), 506968735 (*Coptotermes formosanus*). Alignment was colored using ESPript [43]. Secondary structure elements from human NUDT16 structure are reported above the alignment. Residues involved in metal, Pα and inosine-binding in the human NUDT16 structure are indicated by an orange circle, a blue triangle and a green square, respectively. The NUDIX motif is boxed in light violet.
(TIF)

S3 Fig Activity assays of NUDT16 towards ITP, XDP and ADP (Michaelis-Menten plots). The graphs were produced by GraphPad Prism (version 5.01 for Windows, GraphPad Software, San Diego California USA, www.graphpad.com) after plotting the initial velocity of the reaction against the concentration of substrate. The regression model used was the Michaelis-Menten equation. Each point corresponds to the average of triplicate measurements, the error bars representing the mean error among these triplicates.
(TIF)

S4 Fig Comparison between the binding-mode of IMP, GTP and m^7^GpppA. (A) Overlay of X29 bound to GTP (PDB ID: 2A8S, colored in salmon) and to m^7^GpppA (PDB ID: 2A8T, colored in white). The nucleotides are shown as sticks with their carbon atoms colored either in yellow (GTP) or green (m^7^GpppA). The Mn^2+^ ions are displayed as violet (complex with GTP) or orange (complex with m^7^GpppA) spheres. Residues interacting directly or through metal interactions are shown as sticks and colored as their respective protein. Interactions are represented by violet (complex with GTP) or orange (complex with m^7^GpppA) dashed lines. (B) Overlay of NUDT16 bound to IMP (cyan) and X29 bound to GTP (PDB: 2A8S, colored in salmon). The nucleotides are shown as sticks with their carbon atoms colored either in green (IMP) or yellow (GTP). The Mn^2+^ ions from X29 structures are displayed as violet spheres while the Mg^2+^ ions present in NUDT16 are shown as orange spheres. Residues interacting with metals, phosphate moieties or the putative catalytic base are shown as sticks and colored as their respective protein. Interactions are represented by green (NUDT16) or yellow (X29) dashed lines.
(TIFF)

S5 Fig Inhibition by IMP of NUDT16 activity towards GDP. All points correspond to the average of triplicates, the error bar representing the mean error among the triplicates. The curve was fitted using a one-site model of inhibition in GraphPad Prism (version 5.01 for Windows, GraphPad Software, San Diego California USA, www.graphpad.com), the top and bottom values being constrained to 100% and 0%, respectively.
(TIFF)

S1 Table Comparison of kinetic parameters of NUDT16 A22V and wild-type towards ITP and XDP. *K*_m_, *k*_cat_ and *k*_cat_/*K*_m_ were determined by fitting the experimental kinetic data to a Michaelis-Menten model in GraphPad Prism version 5.01 for Windows, GraphPad Software, San Diego California USA, www.graphpad.com. Values corresponding to NUDT16 wild-type are the ones presented in Table 1.
(DOCX)

S1 Text Supplementary Material and Methods. Description of the experimental procedures used to *i)* express and purify NUDT16 wild-type and A22V mutant for analysis by gel filtration and activity assay, respectively and *ii)* determine the oligomeric state of NUDT16 wild-type in gel filtration.
(DOCX)
