# Supplementary figures and images for: Structural Basis for the Specificity of Human NUDT16 and Its Regulation by Inosine Monophosphate
Source: PLoS One. 2015 Jun 29;10(6):e0131507. doi: 10.1371/journal.pone.0131507 (PMC4485890; doi:10.1371/journal.pone.0131507)

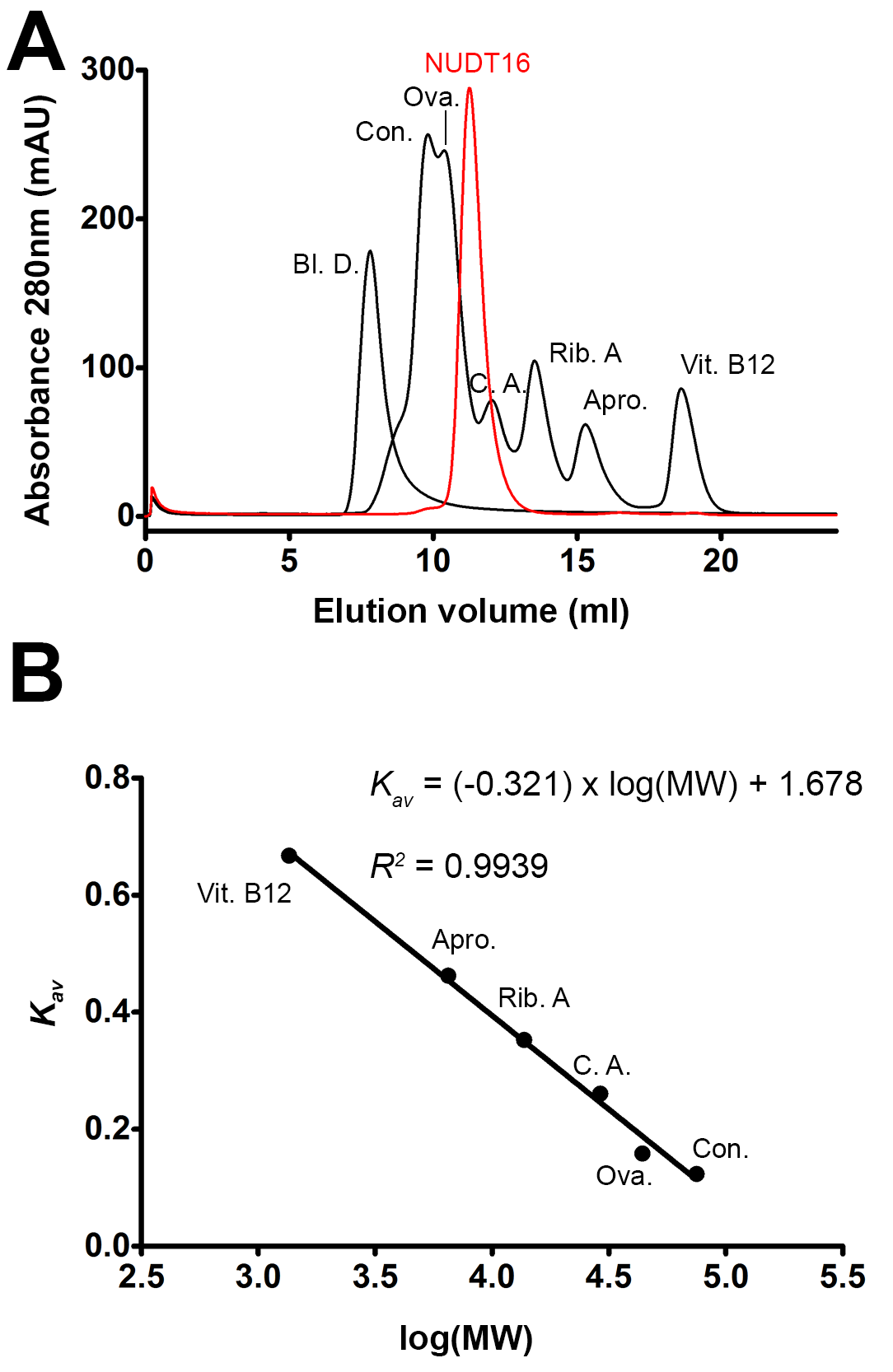

Supplement: S1 Fig — (A) Overlay of gel filtration profiles. Protocol and elution volumes are presented in S1 Text. The chromatograms corresponding to standard proteins and NUDT16 are colored in black and red, respectively. The macromolecules used to generate the standard curve are abbreviated as such: Vit. B12 (vitamin B12), Apro. (aprotinin), Rib. A (ribonuclease A), C. A. (carbonic anhydrase), Ova. (ovalbumin), Con. (conalbumin), Bl. D. (blue dextran). (B) Calibration curve. The procedure used to determine the calibration curve and its equation is described in S1 Text. The equation obtained by linear regression and the coefficient of determination, R 2, are indicated in the graph area. The same abbreviations as in S1A Fig are used to identify the K av values of the macromolecules used for the calibration. (TIF) [file pone.0131507.s001.tif]

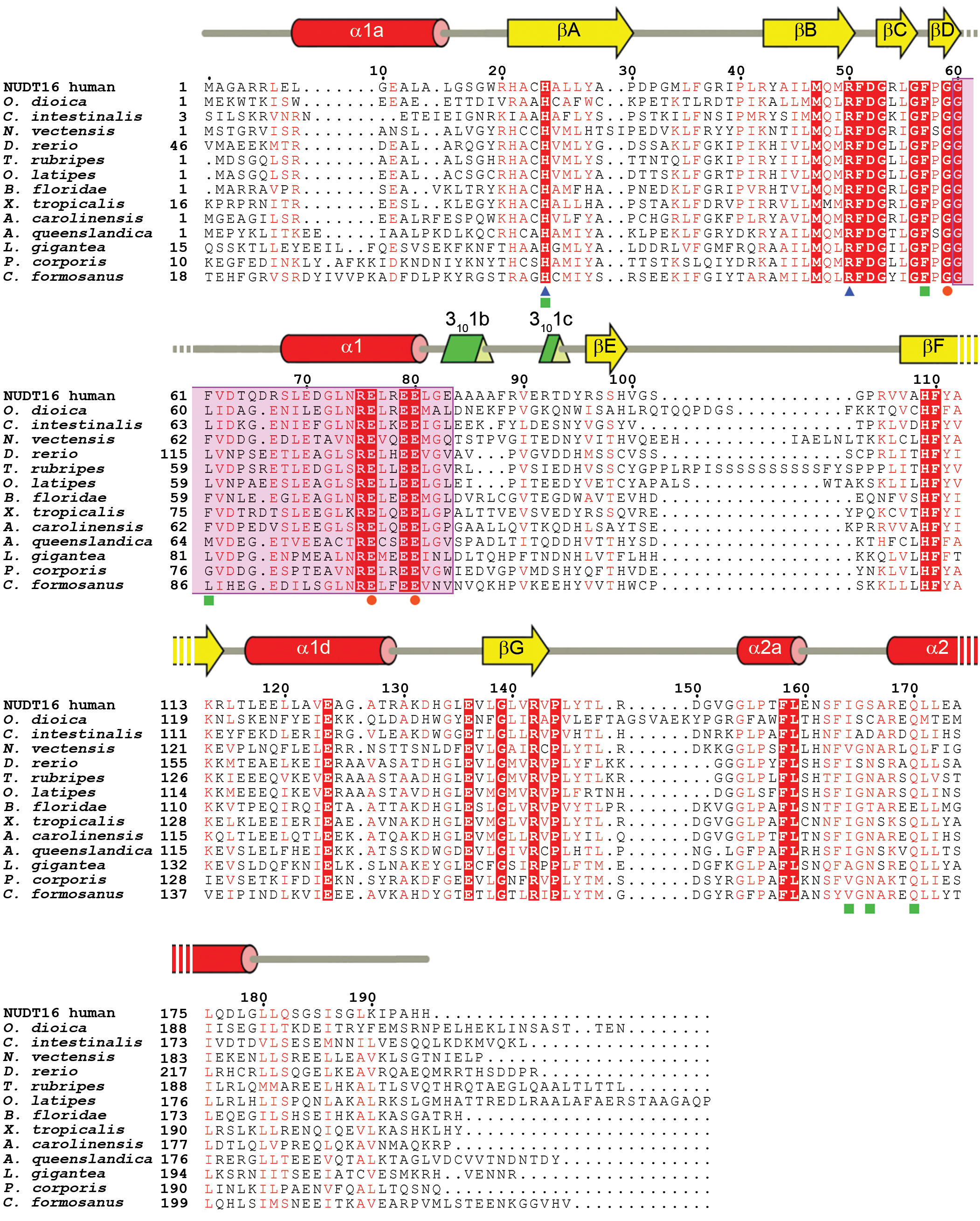

Supplement: S2 Fig — From the top to the bottom, the sequences used in the alignment correspond to the following GenBank IDs: 285026434 (Homo sapiens), 313231008 (Oikopleura dioica), 198435502 (Ciona intestinalis), 156393910 (Nematostella vectensis), 528517610 (Danio rerio), 410898918 (Takifugu rubripes), 432864527 (Oryzias latipes), 260810044 (Branchiostoma floridae), 284813508 (Xenopus (Silurana) tropicalis), 327264231 (Anolis carolinensis), 340368437 (Amphimedon queenslandica), 556107045 (Lottia gigantean), 242004576 (Pediculus humanus corporis), 506968735 (Coptotermes formosanus). Alignment was colored using ESPript [43]. Secondary structure elements from human NUDT16 structure are reported above the alignment. Residues involved in metal, Pα and inosine-binding in the human NUDT16 structure are indicated by an orange circle, a blue triangle and a green square, respectively. The NUDIX motif is boxed in light violet. (TIF) [file pone.0131507.s002.tif]

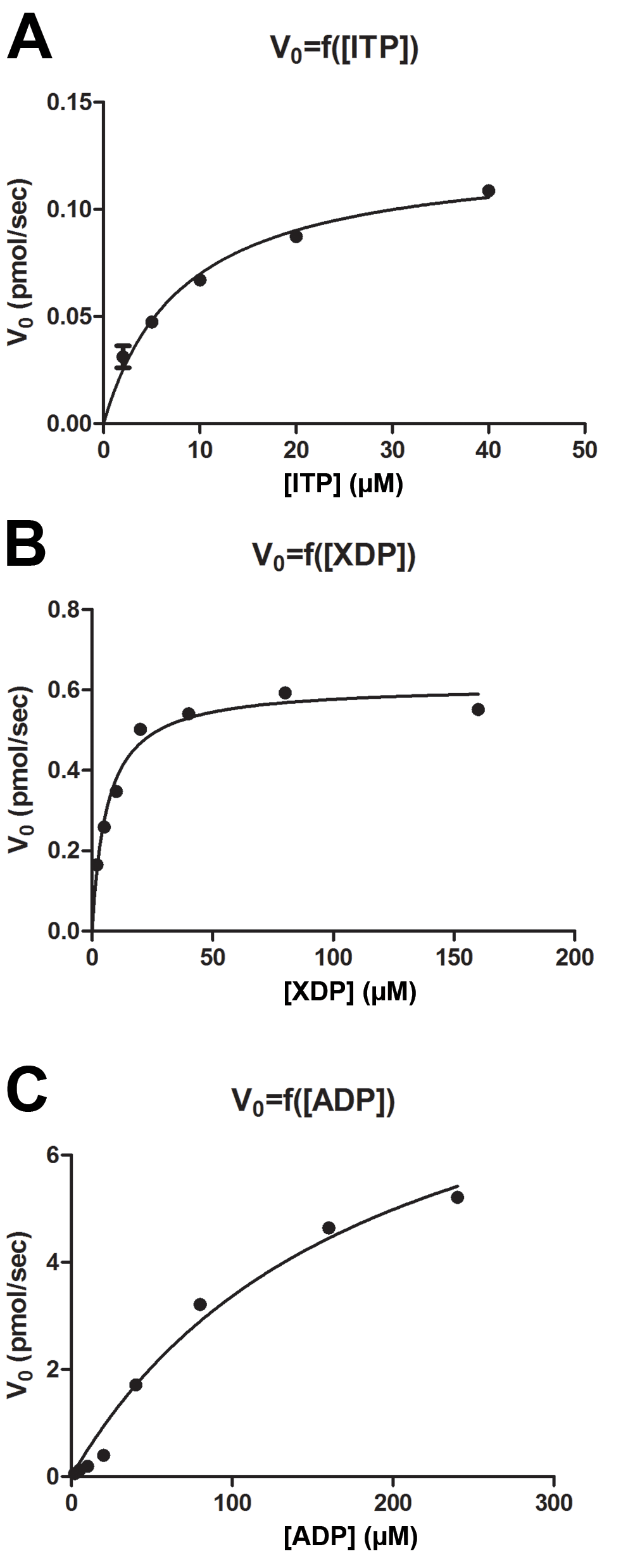

Supplement: S3 Fig — The graphs were produced by GraphPad Prism (version 5.01 for Windows, GraphPad Software, San Diego California USA, www.graphpad.com) after plotting the initial velocity of the reaction against the concentration of substrate. The regression model used was the Michaelis-Menten equation. Each point corresponds to the average of triplicate measurements, the error bars representing the mean error among these triplicates. (TIF) [file pone.0131507.s003.tif]

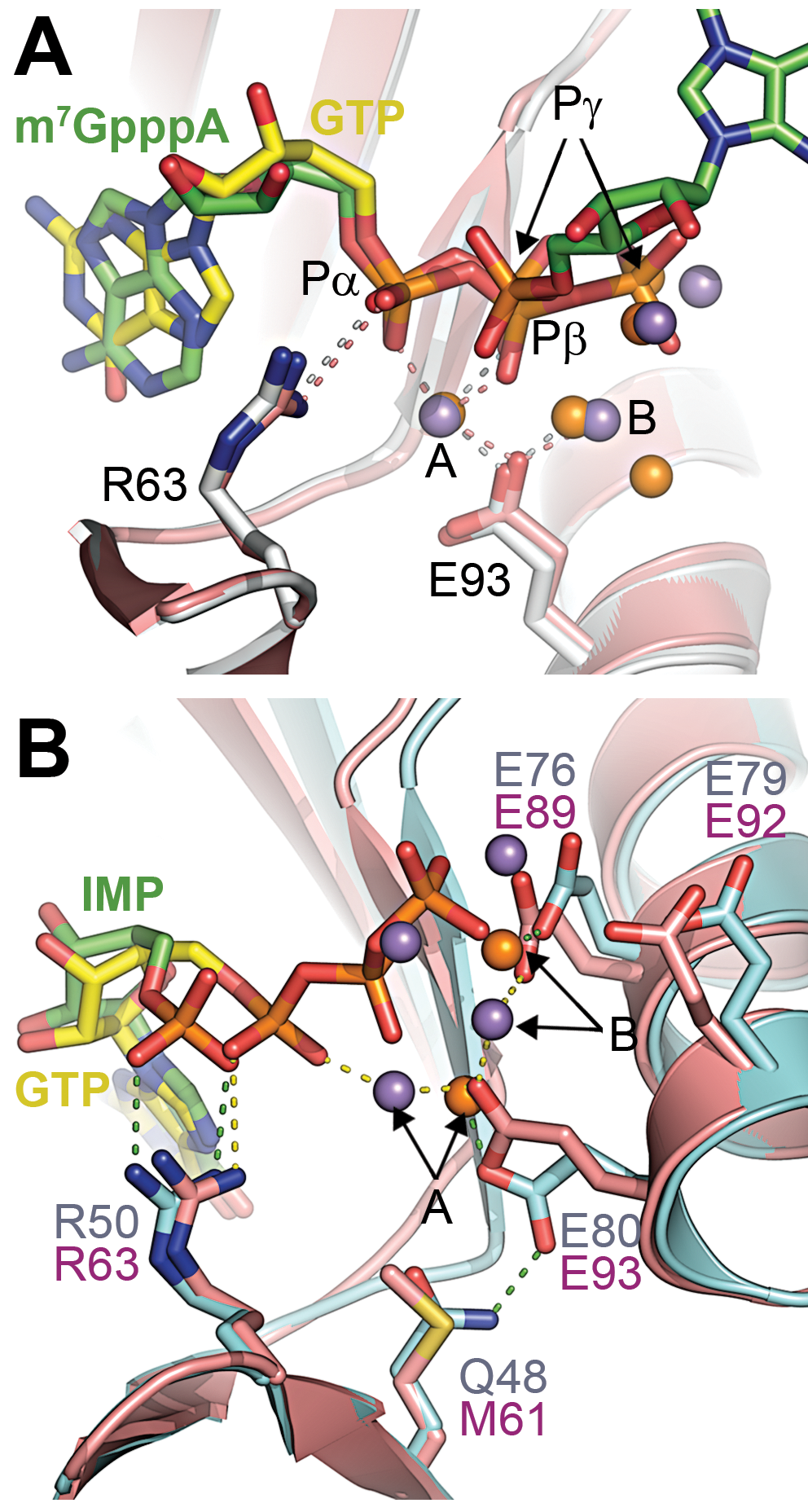

Supplement: S4 Fig — (A) Overlay of X29 bound to GTP (PDB ID: 2A8S, colored in salmon) and to m7GpppA (PDB ID: 2A8T, colored in white). The nucleotides are shown as sticks with their carbon atoms colored either in yellow (GTP) or green (m7GpppA). The Mn2+ ions are displayed as violet (complex with GTP) or orange (complex with m7GpppA) spheres. Residues interacting directly or through metal interactions are shown as sticks and colored as their respective protein. Interactions are represented by violet (complex with GTP) or orange (complex with m7GpppA) dashed lines. (B) Overlay of NUDT16 bound to IMP (cyan) and X29 bound to GTP (PDB: 2A8S, colored in salmon). The nucleotides are shown as sticks with their carbon atoms colored either in green (IMP) or yellow (GTP). The Mn2+ ions from X29 structures are displayed as violet spheres while the Mg2+ ions present in NUDT16 are shown as orange spheres. Residues interacting with metals, phosphate moieties or the putative catalytic base are shown as sticks and colored as their respective protein. Interactions are represented by green (NUDT16) or yellow (X29) dashed lines. (TIFF) [file pone.0131507.s004.tiff]

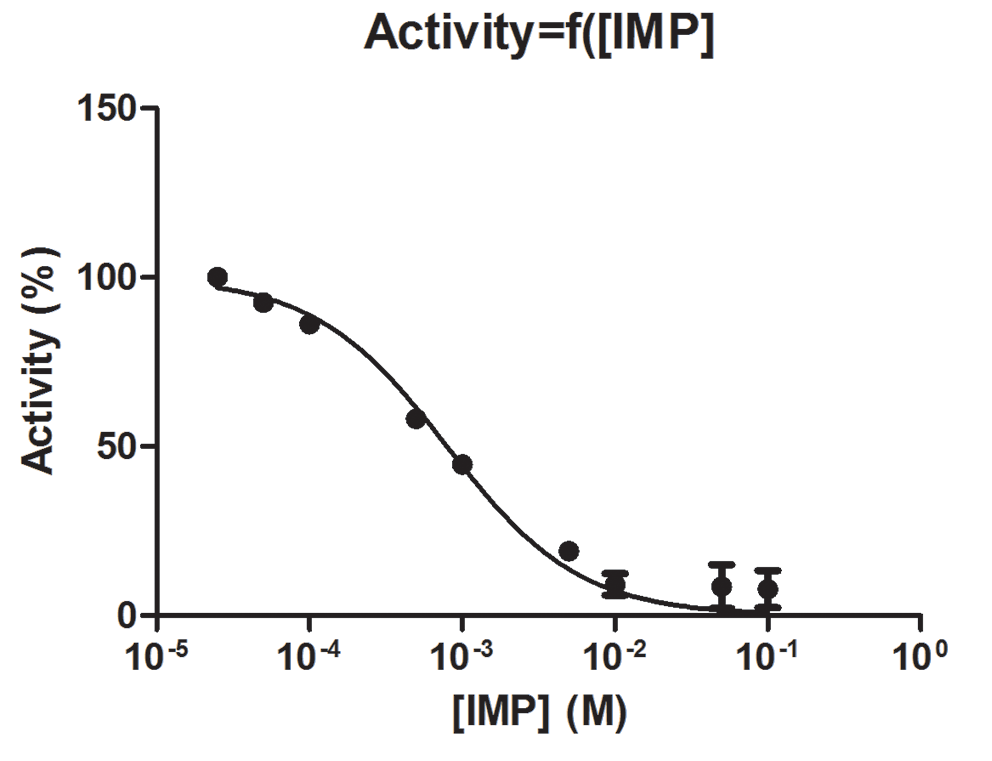

Supplement: S5 Fig — All points correspond to the average of triplicates, the error bar representing the mean error among the triplicates. The curve was fitted using a one-site model of inhibition in GraphPad Prism (version 5.01 for Windows, GraphPad Software, San Diego California USA, www.graphpad.com), the top and bottom values being constrained to 100% and 0%, respectively. (TIFF) [file pone.0131507.s005.tiff]
