# Supplementary material for: Structural Basis for the Specificity of Human NUDT16 and Its Regulation by Inosine Monophosphate
Source: PLoS One. 2015 Jun 29;10(6):e0131507. doi: 10.1371/journal.pone.0131507 (PMC4485890; doi:10.1371/journal.pone.0131507)
Supplement: S1 Table — K m, k cat and k cat/K m were determined by fitting the experimental kinetic data to a Michaelis-Menten model in GraphPad Prism version 5.01 for Windows, GraphPad Software, San Diego California USA, www.graphpad.com. Values corresponding to NUDT16 wild-type are the ones presented in Table 1. (DOCX) [file pone.0131507.s006.docx]

|  | *K*_m_ (µM) | | *k*_cat_ (min^-1^) | | *k*_cat_/*K*_m_ (10^3^ s^-1^M^-1^) | |
| --- | --- | --- | --- | --- | --- | --- |
| Substrate | NUDT16 wild-type | NUDT16 A22V | NUDT16 wild-type | NUDT16 A22V | NUDT16 wild-type | NUDT16 A22V |
| ITP | 8.23 ± 1.01 | 12.82 ± 1.46 | 0.764 ± 0.030 | 0.394 ± 0.013 | 1.55 ± 0.20 | 0.513 ± 0.06 |
| XDP | 6.22 ± 0.55 | 9.70 ± 1.25 | 0.368 ± 0.078 | 0.309 ± 0.012 | 0.99 ± 0.23 | 0.53 ± 0.07 |
