# Supplementary material for: Structural Basis for the Specificity of Human NUDT16 and Its Regulation by Inosine Monophosphate
Source: PLoS One. 2015 Jun 29;10(6):e0131507. doi: 10.1371/journal.pone.0131507 (PMC4485890; doi:10.1371/journal.pone.0131507)
Supplement: S1 Text — Description of the experimental procedures used to i) express and purify NUDT16 wild-type and A22V mutant for analysis by gel filtration and activity assay, respectively and ii) determine the oligomeric state of NUDT16 wild-type in gel filtration. (DOCX) [file pone.0131507.s007.docx]

**S1 Text. Supplementary Material and Methods.**

## Expression and purification of NUDT16 wild-type (for analytical gel-filtration) and NUDT16 A22V (for activity assays)

The protocol was identical for both NUDT16 wild-type and NUDT16 A22V. Expression and purification procedures were similar to the ones described in the section “Cloning, expression and purification of NUDT16 for functional studies (isothermal titration calorimetry and activity assays)” under “Material and Methods” in the main text, with the following exceptions: the bacterial strain was *E. coli* Rosetta; the cultures were grown in a total volume of 1.4 l of TB in TunAir® flasks; the affinity purification step was performed in a gravity flow column loaded with 6 ml of Ni-NTA agarose resin (Invitrogen™) equilibrated in buffer A (20 mM Hepes pH 7.5, 500 mM NaCl, 10% glycerol, 10 mM imidazole and 0.5mM TCEP). Bound NUDT16 was washed first in 20 column volumes of buffer A, then in 20 column volumes of buffer A containing 25 mM imidazole instead of 10 mM. 1.38 mg of TEV protease was added to the resin and cleavage occurred overnight at 4 C. The resin was then washed with 3 times 10 ml of buffer A supplemented with 25 mM imidazole. Fractions containing cleaved proteins were pooled and concentrated in a Vivaspin 20 (Sartorius) (cut-off 10000Da) to a final volume of 2 ml.

Samples were then injected onto a Superdex S200 HiLoad 16/60 (GE Healthcare) equilibrated in buffer B (20 mM Hepes pH 7.5, 300 mM NaCl, 10% glycerol and 0.5 mM TCEP). Eluted fractions containing NUDT16 (as verified by SDS-PAGE analysis) were pooled together and concentrated in a Vivaspin 20 (Sartorius) (cut-off 10000Da) until the protein concentration reached 17.3 mg/ml (NUDT16 wild-type) or 11.8 mg/ml (NUDT16 A22V). Samples were then frozen in liquid nitrogen and stored at -80°C.

## Analytical gel filtration

A Superdex 75 10/300 GL (GE Healthcare) was equilibrated in 50 mM Na_2_HPO_4_/NaH_2_PO_4_, 150 mM NaCl (analytical gel filtration buffer), pH 7.0. 100 µl of a mix composed with 2.2 mg/ml conalbumin (MW 75000 Da), 2.2 mg/ml ovalbumin (MW 44000 Da), 2.2 mg/ml carbonic anhydrase (MW 29000 Da), 4.5 mg/ml ribonuclease A (MW 13700 Da), 1.8 mg/ml aprotinin (MW 6500 Da) and 0.1 mg/ml vitamin B12 (MW 1355 Da) in analytical gel filtration buffer are injected onto the column. In the two next runs, 100µl of a 1 mg/ml blue dextran solution then 100 µl of a 5 mg/ml NUDT16 wild-type solution in analytical gel filtration buffer are injected onto the column. In all cases, elution is performed in analytical gel filtration buffer at 0.4 ml/min.

These are the elution volumes we determined for the different macromolecules: vitamin B12, 18.62 ml; aprotinin, 15.30 ml; ribonuclease A, 13.52 ml; carbonic anhydrase, 12.03 ml; ovalbumin, 10.38 ml; conalbumin, 9.81 ml; blue dextran, 7.81 ml; NUDT16 wild-type, 11.26 ml.

Elution volumes are used to determine *K_av_* using the following formula: where Ve is the elution volume of the standard proteins, V_0_ the elution volume of blue dextran and V_t_ the bed volume of the column (24 ml).

GraphPad Prism version 5.01 for Windows (GraphPad Software, San Diego California USA, [www.graphpad.com](http://www.graphpad.com)) was used to perform the linear regression of *K_av_*=f(log(MW)). With this method, we obtained an approximated value of 36780 Da for the molecular weight of NUDT16 which corresponds to 1.7 times the molecular weight of a monomer.

Results are reported in S1 Fig.
